# Use of DNA methylation patterns for early detection and management of lung cancer: Are we there yet?

**DOI:** 10.32604/or.2024.057231

**Published:** 2025-03-19

**Authors:** MILICA KONTIC, FILIP MARKOVIC

**Affiliations:** 1 Clinic for Pulmonology, University Clinical Center of Serbia, Belgrade, 11000, Serbia; 2 School of Medicine, University of Belgrade, Belgrade, 11000, Serbia

**Keywords:** Lung cancer, DNA methylation, Epigenetics, Hypermethylation, Early detection

## Abstract

Detecting lung cancer early is crucial for improving survival rates, yet it remains a significant challenge due to many cases being diagnosed at advanced stages. This review aims to provide advances in epigenetics which have highlighted DNA methylation patterns as promising biomarkers for early detection, prognosis, and treatment response in lung cancer. Techniques like bisulfite conversion followed by PCR, digital droplet polymerase chain reaction, and next-generation sequencing are commonly used for detecting these methylation patterns, which occur early in the cancer development process and can be detected in non-invasive samples like blood and sputum. Key genes such as SHOX2 and RASSF1A have demonstrated high sensitivity and specificity in clinical studies, making them crucial for diagnostic purposes. However, several challenges remain to be overcome before these biomarkers can be widely adopted for use in clinical practice. Standardizing the assays and validating their effectiveness are critical steps. Additionally, integrating methylation biomarkers with existing diagnostic tools could significantly enhance the accuracy of lung cancer detection, providing a more comprehensive diagnostic approach. Although progress has been made in understanding and utilizing DNA methylation patterns for lung cancer detection, more research and extensive clinical trials are necessary to fully harness their potential. These efforts will help establish the robustness of methylation patterns as biomarkers and therapeutic targets, ultimately leading to better prevention, diagnosis, and treatment strategies for lung cancer. In conclusion, DNA methylation states represent a promising avenue for advancing early detection, accurate diagnosis, and management of lung cancer.

## Introduction

Lung cancer remains the most diagnosed cancer worldwide, accounting for nearly 2.5 million new cases in 2022, or 12.4% of all global cancer diagnoses. It is also the primary cause of cancer-related deaths. In women, lung cancer ranks second in both incidence and mortality. Among men, lung cancer is the most prevalent cancer in terms of both cases and deaths [1]. Unfortunately, approximately 85% of patients with lung cancer are diagnosed when curative treatment is not an option, and the disease is in the advanced stage. This late-stage diagnosis contributes to the poor prognosis and 5-year overall survival rate of only 19% across all stages. With the disease progression from early to advanced stages, the 5-year survival rate declines significantly [2]. Early detection significantly improves prognosis and survival, emphasizing the need for reliable biomarkers that can identify lung cancer at its earliest stages. Traditional diagnostic methods, including imaging and histopathological examination, often fail to detect lung cancer until it has progressed to an advanced stage. Consequently, there is a critical demand for non-invasive, sensitive, and specific biomarkers to facilitate early detection [3].

Epigenetic alterations, particularly DNA methylation, have gained recognition as promising biomarkers for the diagnosis of cancer. DNA methylation involves the addition of a methyl group to the cytosine ring within CpG dinucleotides, typically leading to gene silencing. Aberrant DNA methylation patterns, such as hypermethylation of tumor suppressor genes as well as global hypomethylation, have a pivotal part in tumorigenesis and disease progression [4]. These epigenetic changes occur early in the development of cancer, making them ideal candidates for biomarkers of detection of lung cancer in its early stages.

Studies have identified several genes with altered patterns of DNA methylation that are associated with lung cancer [5,6]. Among these, SHOX2 and RASSF1A have demonstrated significant potential as diagnostic biomarkers [4–7]. SHOX2 methylation has been known to have both high sensitivity and specificity for lung cancer detection, while RASSF1A methylation provides additional diagnostic accuracy when used in combination. Detection methods such as bisulfite conversion followed by polymerase chain reaction (PCR), digital droplet PCR (ddPCR), and next-generation sequencing (NGS) have been employed to analyze these methylation patterns with varying degrees of success.

Despite these advancements, the clinical implementation of DNA methylation biomarkers for lung cancer detection faces several challenges. Assay protocol standardization, validation across different populations as well as integration with diagnostic workflows at hand are necessary steps to ensure reliability and reproducibility. Furthermore, large-scale prospective clinical trials are necessary to establish the utility of these biomarkers in clinical practice.

The present review aims to provide a review of the current achievements in utilizing methylation patterns of DNA for the detection of lung cancer in its early stages. Additionally, it explores the key genes and their methylation status, the methodologies employed for detection, the clinical applications, and the challenges that need to be addressed. Future directions and potential advancements that could enhance the early detection, diagnosis and treatment of lung cancer, ultimately improving outcomes of patients are also examined.

## DNA Methylation and Its Link to Cancer

DNA methylation is an epigenetic modification involving the addition of a methyl group (CH3) to the 5-carbon position of the cytosine ring within CpG dinucleotides. This process is catalyzed by DNA methyltransferases (DNMTs). In normal cells, DNA methylation plays a crucial role in regulating gene expression, maintaining genomic stability, and silencing repetitive elements [3].

**Figure 1:**
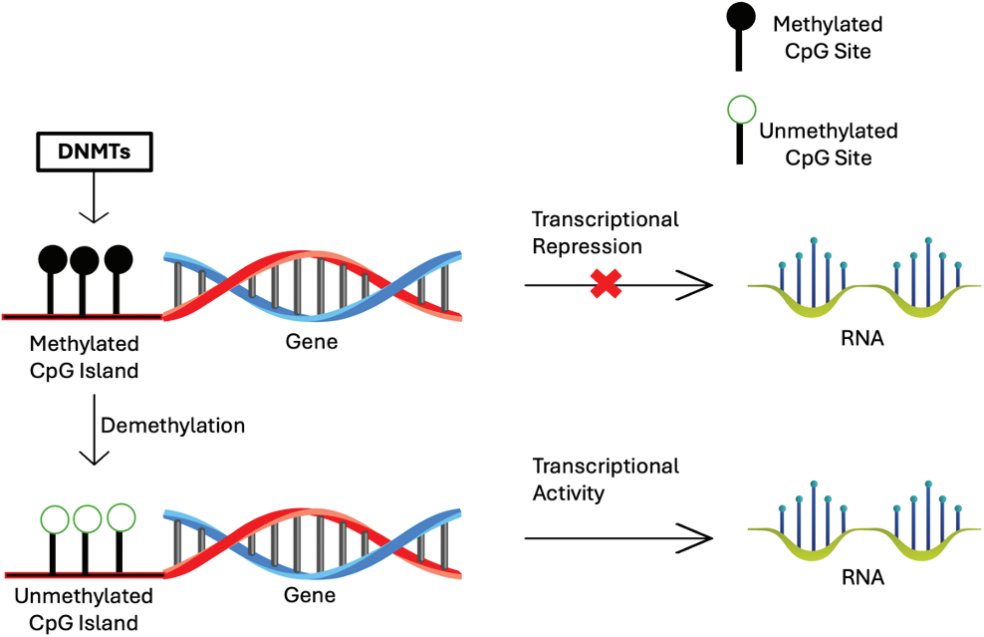
Scheme of the process of DNA methylation regulated the gene expression.

In cancer, DNA methylation patterns are often disrupted, leading to aberrant gene expression [8] (Fig. 1).

There are two primary types of DNA methylation changes that are associated with cancer: hypermethylation and hypomethylation. These epigenetic changes are stable and occur early in the process of carcinogenesis, making them ideal biomarkers for early cancer detection [3,7–9].

Hypermethylation commonly occurs at CpG islands in the promoter regions of tumor suppressor genes. This abnormal addition of methyl groups can silence tumor suppressor genes, preventing them from controlling cell growth and division. Consequently, the silencing of these genes contributes to uncontrolled cellular proliferation and tumor development.

On the other hand, hypomethylation generally occurs across the genome and is characterized by a reduction in the normal methylation levels. This can lead to several oncogenic effects—the activation of oncogenes, which are genes that promote cell growth and division. When these genes become improperly activated, they can contribute to cancer development and progression. Additionally, hypomethylation causes genomic instability. Reduced methylation levels lead to chromosomal instability and an increased mutation rate that drives cancer progression.

## Early Detection of Lung Cancer-DNA Methylation as a Biomarker

DNA methylation patterns of specific genes can serve as biomarkers for the detection of lung cancer in its early stages. Hypermethylation of specific genes can be detectable in non-invasive samples such as blood and sputum, offering a less invasive alternative to traditional biopsy methods and providing promising diagnostic options. Studies have demonstrated that the methylation of genes like CDKN2A (p16), MGMT, and RASSF1A can be identified in sputum samples from high-risk individuals before clinical diagnosis of lung cancer. For example, methylation of the CDKN2A gene was detected in 100% of sputum samples from individuals who were later diagnosed with squamous cell carcinoma, with a lead time of up to three years before clinical diagnosis [10,11].

The use of sputum to detect hypermethylation of these specific genes leverages the presence of exfoliated respiratory tract cells that could be analyzed for epigenetic changes. This method has shown an increase in the prevalence of DNA methylation as the time to lung cancer diagnosis shortens [7,9]. This early detection capability underscores the potential of sputum-based methylation assays for screening high-risk populations, such as smokers.

Furthermore, combining multiple hypermethylated genes into a panel can enhance diagnostic sensitivity and specificity. For instance, detecting methylation in three or more genes was associated with a 6.5-fold increase in lung cancer risk, with a sensitivity and specificity of around 64% [10].

Blood-based detection, specifically using circulating tumor DNA (ctDNA), is another promising approach. ctDNA can carry methylation markers that reflect the epigenetic changes occurring in tumors. Techniques like methylation-specific PCR (MSP), ddPCR, and NGS are used to analyze ctDNA for methylation patterns. These methods offer high specificity and sensitivity, enabling lung cancer diagnosis in its early stage. For instance, methylation of genes such as SHOX2 and RASSF1A in plasma has been associated with lung cancer in its early stages, demonstrating the potential of blood-based assays for non-invasive screening [12,13].

These findings support the use of gene promoter hypermethylation in non-invasive samples as a molecular marker for early lung cancer detection. Specimens that can be used for early detection using DNA methylation markers are sputum and ctDNA. Sputum may be a more useful specimen for cancers occurring in the bronchial trees compared to ctDNA. On the other hand, in the case of ctDNA, DNA methylation markers could be detected regardless of the anatomical location associated with the tissue type of lung cancer.

Further research is crucial however in order to standardize assays and validate their efficacy among the diverse population of patients, ultimately integrating them into routine clinical practice for improved patient outcomes.

## Differential Expression of DNMTs and DNA Methylation Hallmarks in Lung Cancer Subtypes

Lung cancer is a diverse disease characterized by various histological as well as molecular subtypes, each exhibiting unique profiles of DNA methylation and associated epigenetic modifications. The DNMTs and other key markers of DNA methylation can vary significantly across histology subtypes, influencing tumor behavior and treatment response.

Lung adenocarcinoma is the predominant histological subtype of non-small cell lung cancer (NSCLC), often exhibits global hypomethylation accompanied by region-specific hypermethylation [14]. DNMT1, DNMT3A, and DNMT3B, which are critical for establishing patterns of DNA methylation, are frequently upregulated among adenocarcinomas. This upregulation contributes to the silencing of tumor suppressor genes through promoter hypermethylation and genomic instability through global hypomethylation [15,16]. Additionally, mutations in genes regulating DNA methylation, such as TET2, have been implicated in adenocarcinoma progression [17,18].

Lung squamous cell carcinoma (SCC) displays a different DNA methylation pattern when compared to adenocarcinoma. SCC often exhibits a distinct DNA methylation signature, characterized by the hypermethylation of certain genes that play a role in the differentiation of cells and regulation of their growth [19]. DNMT3A and DNMT3B are frequently upregulated in SCC, facilitating the silencing of specific genes such as CDKN2A and RASSF1A [20]. Global hypomethylation is also observed, contributing to chromosomal instability.

Large cell carcinoma (LCC) is another subtype of NSCLC with a less well-defined DNA methylation profile compared to adenocarcinoma and SCC. However, studies suggest that LCC often exhibits alterations in DNMT expression and DNA methylation patterns similar to those seen in adenocarcinoma, albeit less pronounced [21]. DNMT1 and DNMT3A are generally upregulated, contributing to the aberrant hypermethylation of certain genes that are involved in both tumor suppression and resistance to drugs [22].

Small cell lung cancer (SCLC) is a type of neuroendocrine tumor with a unique DNA methylation profile. SCLC tumors frequently exhibit extensive global hypomethylation and hypermethylation of specific tumor suppressor genes [23]. DNMT1 is commonly overexpressed, which is associated with the aberrant DNA methylation of genes such as TP53 and RB1, which are known to play pivotal roles in both maintenance of genomic stability and regulation of the cell cycle progression [24]. This hypermethylation contributes to the aggressive nature and high metastasis rates observed in SCLC.

Carcinoid tumors, particularly atypical carcinoids, show distinct DNA methylation patterns compared to other lung cancer subtypes. They often have lower levels of DNMT expression, and thus a lower degree of global DNA methylation changes. However, specific gene hypermethylation, such as of the MGMT and RASSF1A genes, has been reported. This suggests a more targeted epigenetic silencing mechanism in these tumors compared to the more widespread changes seen in other lung cancer subtypes [25,26].

The differential expression of DNMTs and the varying patterns of DNA methylation across lung cancer subtypes reflect their distinct biological behaviors and clinical outcomes. Understanding these variations could help us to better understand the process of lung cancer pathogenesis in addition to serving as potential targets for subtype-specific epigenetic therapies.

## Key Methylation Biomarkers in Lung Cancer

In the following section, we will discuss the most extensively studied DNA methylation biomarkers in lung cancer (Table 1).

**Table 1:** Key methylation markers according to histological types of lung cancer

DNA methylation marker	Tumor type
SHOX2	Adenocarcinoma
	SCC
	SCLC
RASSF1A	Adenocarcinoma
	Carcinoid
CDKN2A (p16)	SCC
	Adenocarcinoma
MGMT	Adenocarcinoma
	SCC
	Large cell carcinoma Carcinoid
APC	Adenocarcinoma
	SCC

**SHOX2:** The methylation status of SHOX2 gene is a well-established biomarker for lung cancer detection [4,6,7]. Hypermethylation of SHOX2 is frequently observed in lung cancer tissues and can be detected in body fluids such as blood and sputum. Several studies have demonstrated the high sensitivity and specificity of SHOX2 methylation for lung cancer diagnosis. One study used the LungMe® assay to assess the methylation levels of SHOX2 and RASSF1A in broncho-exfoliated cells, demonstrating high diagnostic accuracy for lung cancer with an area under the curve (AUC) of 0.814, indicating excellent sensitivity and specificity for detecting lung cancer through bronchoscopic samples [4,6]. Methylation of the SHOX2 gene has been associated with early stages of lung cancer, providing a crucial tool for early diagnosis.

Another study showed that the pooled sensitivity and specificity of SHOX2 DNA methylation were 70% and 96%, respectively [6,7]. These studies show that HOX2 methylation has higher diagnostic accuracy compared to traditional serum-based biomarkers, such as cytokeratin 19 fragment antigen (CYFRA 21-1) and carcinoembryonic antigen (CEA) [7].

Additionally, the performance of SHOX2 methylation in formalin-fixed, paraffin-embedded (FFPE) samples was evaluated, showing a sensitivity of 78.1% and a specificity of 92.1% in distinguishing lung cancer from benign lesions [5]. This study also found that combining SHOX2 with RASSF1A methylation further improved diagnostic accuracy [5].

These studies collectively suggest that SHOX2 methylation is a robust biomarker for the early detection of lung cancer, with several demonstrating high sensitivity and specificity in various clinical sample types.

**RASSF1A:** The RASSF1A gene is another important tumor suppressor gene that has been known to have important roles in cell cycle regulation as well as apoptosis [27]. It is frequently hypermethylated in lung cancer which results in gene silencing, contributing to tumorigenesis. Methylation of RASSF1A could be detected in ctDNA in the blood, providing a non-invasive method for early detection [10,12]. The correlation of this marker with disease stage and prognosis has been shown.

**CDKN2A (p16):** The CDKN2A gene, known as p16, is yet another tumor suppressor gene involved in the process of cell cycle regulation [28]. Its promoter hypermethylation leads to gene silencing, preventing it from controlling cell proliferation. In lung cancer, CDKN2A hypermethylation is common and has been detected in sputum samples years before clinical diagnosis, highlighting its potential as an early detection biomarker [9,10]. The presence of methylated CDKN2A in non-invasive samples suggests its role in identifying high-risk individuals even before the onset of clinical symptoms.

**MGMT:** The MGMT gene is involved in DNA repair. Hypermethylation of its promoter has been linked with gene silencing in solid neoplasms including lung cancer [29]. Detection of MGMT methylation in sputum and plasma has shown promise as a non-invasive biomarker for the detection of lung cancer in its early stages and risk assessment. It has been shown that MGMT hypermethylation is present in a significant proportion of sputum samples from individuals who later develop lung cancer, underscoring its utility in early screening programs [10,11].

**Adenomatous Polyposis Coli (APC):** APC is yet another tumor suppressor gene with significant roles in the regulation of signaling pathways as well as cell adhesion [30]. Hypermethylation of the APC promoter leads to gene silencing, promoting cancer development. APC methylation was evident in samples of sputum from lung cancer patients, emphasizing the potential of APC methylation as an early detection biomarker [11,12]. The ability to detect APC methylation in non-invasive samples could significantly enhance early screening efforts.

## Methods for Detection of Methylation Biomarkers

To detect global DNA hypomethylation several methodologies can be employed, focusing on comprehensively assessing methylation across the genome:

**Bisulfite Conversion and PCR:** The most common method for detecting DNA methylation involves bisulfite conversion, which converts unmethylated cytosines to uracils while leaving methylated cytosines unchanged. This is followed by PCR to amplify the regions of interest. MSP and quantitative MSP (qMSP) are widely used techniques. MSP differentiates between methylated and unmethylated DNA after bisulfite treatment. It is highly sensitive and specific, making it suitable for detecting methylated DNA even in low quantities in patient samples [12,13].

**NGS:** NGS-based methods allow for the comprehensive analysis of methylation patterns across the genome. Techniques such as whole-genome bisulfite sequencing (WGBS) and targeted bisulfite sequencing provide high-resolution maps of DNA methylation and can identify novel biomarkers. Pyrosequencing provides quantitative analysis of DNA methylation and is particularly useful for examining the methylation status of individual CpG sites within a gene [12,13]. It involves real-time sequencing and provides accurate methylation levels

**Digital PCR (dPCR) and ddPCR:** These methods partition the DNA sample into thousands of droplets or wells, allowing for precise quantification of methylated DNA molecules. ddPCR is an advanced method that enhances the sensitivity and specificity of methylation detection. It allows for the absolute quantification of methylated DNA molecules, making it suitable for detecting rare methylation events in liquid biopsies [12].

**BeadArray Technology:** The Illumina Infinium MethylationEPIC BeadChip is a high-throughput platform that can analyze over 850,000 CpG sites simultaneously. This technology is used for large-scale DNA methylation studies and can identify potential biomarkers for early cancer detection.

**5-methylcytosine (5-mC) Immunofluorescence Assays:** The process involves using antibodies specific to 5-mC to stain cells or tissue sections, which can then be analyzed by fluorescence microscopy. This method provides a visual representation of DNA methylation patterns within cells and can be quantified using image analysis software to assess global DNA methylation levels.

**High-Resolution Image Cytometry:** This approach utilizes advanced imaging technologies combined with cytometry to provide detailed information about the topology of DNA methylation across individual cells. High-resolution image cytometry can measure the intensity and distribution of methylation-specific fluorescence within a cell, offering insights into global DNA methylation landscapes at the single-cell level.

**Nanotechnologies:** There have been significant advancements that have facilitated the utilization of nanotechnology for DNA methylation-based detection, particularly in the context of lung cancer diagnostics. One notable development involves the use of DNAzyme-assisted aptasensors [31]. These aptasensors leverage gold nanorods and electroluminescent materials to enhance the detection sensitivity for cancer biomarkers like carcinoembryonic antigen (CEA), achieving detection limits in the attogram range [31]. This indicates a substantial improvement in the capability to detect minute quantities of DNA methylation associated with cancer, which is crucial for early diagnosis and treatment.

### Assessing global DNA methylation: cell-based immunofluorescence and high-resolution image cytometry

Global DNA methylation, particularly hypomethylation, plays a crucial role in genomic stability and gene expression regulation, marking its relevance as a biomarker for various pathological conditions, including cancer. Traditional methods for assessing DNA methylation typically provide bulk population data, which may obscure critical differences among individual cells.

Cell-based immunofluorescence assays involve staining cells with antibodies specific to 5-methylcytosine. This technique allows for the visualization of DNA methylation patterns directly within cells, using fluorescence microscopy. By employing these assays, it is possible to detect and quantify the levels of DNA methylation in individual cells, providing insights into the heterogeneity of methylation status within a cell population [32].

High-resolution image cytometry extends the capabilities of traditional flow cytometry by combining it with advanced imaging techniques. This method enables the detailed examination of the spatial distribution and quantification of DNA methylation within single cells. The topology of DNA methylation across the cell nucleus can be analyzed, offering valuable data on global DNA methylation patterns and identifying aberrant cells with altered methylation states [33,34].

The integration of cell-based immunofluorescence assays and high-resolution image cytometry provides a robust platform for the detailed investigation of global DNA hypomethylation at the single-cell level. This approach addresses key challenges in identifying aberrant cells and quantifying DNA methylation, which is crucial for early diagnosis, risk assessment, and monitoring of disease progression in cancer and other diseases marked by epigenetic alterations [35–37].

The combined use of these sophisticated methodologies enables a more nuanced understanding of epigenetic modifications and their implications in disease pathogenesis, advancing the potential for personalized medicine and targeted therapeutic strategies.

This single-cell approach is essential for advancing our understanding of epigenetic regulation and its impact on cell function and disease, offering a more precise and comprehensive analysis of DNA methylation than traditional methods.

### Sources of specimen for early detection of lung cancer using DNA methylation markers

In studies focusing on the detection of lung cancer in its early stages using DNA methylation markers, different biomarkers have been identified for use with different specimen types like sputum and ctDNA.

For sputum samples, genes such as MGMT, CDKN2A, DAPK, RASSF1A, PAX5β, and GATA5 have been identified as key DNA methylation markers. For instance, MGMT and CDKN2A hypermethylation has been frequently detected in sputum samples from patients who eventually developed SCC of the lung, with detection preceding the diagnosis of lung cancer by up to three years. On the other hand, the levels of global DNA hypomethylation measured in exfoliated epithelial cells in sputum samples also serve as a differential biomarker across histological subtypes of lung cancer. The sensitivity of detecting lung cancer through sputum-based DNA methylation assays has been reported to be significant, suggesting their utility in population-based screenings for lung cancer, especially in high-risk groups [10,36–38].

For ctDNA, a variety of methylation markers have been studied, showing potential for early detection and even aiding in stratifying lung cancer. Studies have utilized panels of differentially methylated regions (DMRs) that include markers like SHOX2 and genes from the HOX family, which have demonstrated significant sensitivity in addition to significant specificity, in differentiating lung cancer from benign conditions. Utilization of ctDNA methylation markers offers a noninvasive alternative for lung cancer diagnosis, particularly suitable for early detection and monitoring response to treatment [38].

Both sputum and ctDNA provide valuable avenues for applying the use of DNA methylation biomarkers in the diagnosis of lung cancer, with each having its specific utility based on the proximity of the sample to the primary tumor and the noninvasive nature of sampling, respectively. These developments represent significant advancements in the noninvasive diagnosis and management of lung cancer, emphasizing the importance of continued research and validation of these biomarkers in everyday clinical practice.

The sensitivity of DNA methylation detection from blood or sputum in lung cancer generally increases along with the disease stage. The heightened sensitivity is due to the larger volume of tumor cells and, as a result, the increased presence of ctDNA in the later stages of the disease. This higher ctDNA burden in advanced stages makes it more likely to detect the disease using methylation markers.

For instance, sensitivity rates reported for circulating tumor DNA methylation vary significantly across different stages. In early stages, such as stage I, sensitivity can be as low as 18%, which increases to 43% in stage II, 81% in stage III, and up to 93% in stage IV [39]. These numbers reflect the incremental likelihood of detecting ctDNA as the cancer progresses and sheds more DNA into the bloodstream due to increased tumor burden [39].

Moreover, methods like digital droplet PCR have been highlighted for their higher sensitivity and precision in detecting low levels of ctDNA, which is especially valuable in the detection of cancer in its early stages. This technique allows for quantification of very low concentrations of ctDNA, making it suitable for early diagnosis where the ctDNA levels are typically much lower [39].

Thus, while earlier stages of cancer present a greater challenge for ctDNA detection due to lower ctDNA levels, advancements in DNA methylation analysis and detection technologies are improving the early diagnostic potential of these methods.

## Clinical Applications

The utilization of DNA methylation as a biomarker in lung cancer may have several clinical applications. DNA methylation biomarkers could be incorporated into screening programs in a high-risk population. Such would include individuals with extensive smoking history as well as those with a history of lung cancer in the family. Non-invasive tests based on blood or sputum samples could facilitate regular monitoring and early intervention [3,12]. Additionally, DNA methylation changes occur early in the carcinogenic process, providing a window for early intervention [14,40–42].

DNA methylation biomarkers can complement existing diagnostic methods, improving the accuracy of lung cancer diagnosis. They can be used in conjunction with imaging techniques to confirm suspicious lesions and guide biopsy decisions. DNA methylation patterns can also provide prognostic information, helping to stratify patients based on their risk of disease progression and tailor treatment strategies accordingly. For instance, hypermethylation of the RASSF1A gene has been associated with poor outcomes in NSCLC patients [40,43].

### Epigenetic drugs in lung cancer

Aberrant patterns of DNA methylation could potentially be used as therapeutic targets which could lead to reactivation of previously silenced tumor suppressor genes as well as enhancement of the efficacy of other treatments, such as immunotherapy. Ongoing research is focused on developing novel epigenetic drugs that specifically target the methylation machinery or its regulatory pathways. These include next-generation DNMT inhibitors and drugs targeting histone modifications that work synergistically with DNA methylation changes. Novel DNMT inhibitors with improved specificity and reduced toxicity are being developed. These drugs aim to provide more effective demethylation with a favorable safety profile, making them adequate for the use long-term use in combination therapies [12].

#### Reversing aberrant DNA methylation

Targeting aberrant DNA methylation involves the use of agents that can reverse these modifications, thereby reactivating silenced tumor suppressor genes and inhibiting tumor growth.

DNA methyltransferase inhibitors (DNMTi), such as 5-azacytidine and decitabine, have been employed to reverse methylation and reactivate silenced genes in cancer cells [12,13]. These drugs incorporate into DNA and inhibit DNA methyltransferases, leading to hypomethylation and reactivation of silenced tumor suppressor genes [44]. Clinical trials have shown that decitabine can induce responses among patients who suffer from lung cancer, with ongoing studies investigating optimal dosing and combination strategies [45,46] (Table 2). In lung cancer cell lines, these agents have been shown to demethylate and reactivate genes such as p16, MGMT, and RASSF1A, resulting in reduced cell proliferation and increased apoptosis [47,48]. Animal studies demonstrate that azacitidine reduces lung tumor burden and metastasis in lung cancer models [49]. Conversely, early-phase trials have demonstrated that azacitidine has modest efficacy in NSCLC patients, particularly when combined with other antineoplastic drugs. A phase II study reported that azacitidine, in combination with entinostat, followed by nivolumab showed a clinical benefit in pretreated patients with advanced NSCLC, with an overall response rate (ORR) of 13% and median overall survival (mOS) of 10.2 months [50].

**Table 2:** Recent trials involving DNA methyltransferase inhibitors

NCT number and study title	Study status	Conditions	Interventions	Phases
**NCT02959437** Azacitidine combined with pembrolizumab and epacadostat in subjects with advanced solid tumors (ECHO-206)	TERMINATED	Advanced malignancies	Azacitidine Pembrolizumab Epacadostat	PHASE I
		Metastatic cancer		PHASE II
**NCT02712905** An open-label, dose-escalation/dose-expansion safety study of INCB059872 in subjects with advanced malignancies	TERMINATED	Solid tumors and hematologic malignancy	INCB059872	PHASE I
			All-trans retinoic acid (ATRA) azacitidine nivolumab	PHASE II
**NCT02009436** Azacitidine in treating patients with stage IV or recurrent non-small cell lung cancer	COMPLETED	NSCLC	Azacitidine	PHASE I
**NCT01545947** Study assessing safety, pharmacokinetics and efficacy of CC-223 with either erlotinib or oral azacitidine in advanced non-small cell lung cancer	COMPLETED	NSCLC	Erlotinib	PHASE II
			Azacitidine	
**NCT01281124** Azacitidine in treating patients with previously treated advanced non-small cell lung cancer	COMPLETED	Recurrent and stage IV NSCLC	Azacitidine	PHASE II
**NCT01207726** Azacitidine and entinostat in treating patients with stage I non-small cell lung cancer that has been removed by surgery	TERMINATED	NSCLC	Azacitidine Entinostat	PHASE II
**NCT00387465** Azacitidine and entinostat in treating patients with recurrent advanced non-small cell lung cancer	COMPLETED	Advanced NCLC	Azacitidine Entinostat	PHASE I
				PHASE II
**NCT04631029 Testing the Addition of an Anti-cancer Drug, Entinostat, to the Usual Chemotherapy and Immunotherapy Treatment (Atezolizumab, Carboplatin and Etoposide) for Previously Untreated Aggressive Lung Cancer That Has Spread**	COMPLETED	SCLC	Atezolizumab	PHASE I
			Carboplatin	
			Entinostat	
			Etoposide	
**NCT02909452 Continuation study of entinostat in combination with pembrolizumab in patients with advanced solid tumors**	COMPLETED	Solid tumors including NSCLC	Entinostat Pembrolizumab	PHASE I
**NCT02437136 Ph1b/2 dose-escalation study of entinostat with pembrolizumab in NSCLC with expansion cohorts in NSCLC, melanoma, and colorectal cancer**	COMPLETED	Solid tumors including NSCLC	Entinostat Pembrolizumab	PHASE I
				PHASE II
**NCT01928576 Phase II anti-PD1 epigenetic therapy study in NSCLC**	COMPLETED	NSCLC	Azacitidine Entinostat Nivolumab	PHASE II
			CC-486 300	
**NCT01594398 Study to assess food effect on pharmacokinetics of entinostat in subjects with breast cancer or non-small cell lung cancer**	COMPLETED	NSCLC	Entinostat	PHASE I
			Erlotinib	
			Exemestane	
**NCT00750698 A phase II exploratory study of erlotinib and SNDX-275 in participants with non-small cell lung carcinoma who are progressing on erlotinib**	TERMINATED	NSCLC	Entinostat Erlotinib	PHASE II
**NCT00602030 Study to evaluate erlotinib with or without SNDX-275 (entinostat) in the treatment of patients with advanced NSCLC**	COMPLETED	NSCLC	Entinostat Erlotinib	PHASE I
				PHASE II
**NCT00387465 Azacitidine and entinostat in treating patients with recurrent advanced non-small cell lung cancer**	COMPLETED	NSCLC	Azacitidine	PHASE I
			Entinostat	PHASE II

Another group of epigenetic drugs are histone deacetylase inhibitors (HDACis) which have demonstrated the ability to induce hyperacetylation of histones, leading to transcriptional activation of tumor suppressor genes and inhibition of oncogenes. This results in cell cycle arrest, apoptosis, and differentiation [51,52]. In animal models, HDACis have shown significant tumor growth inhibition and enhanced sensitivity to chemotherapy and radiation [53]. There are three drugs tested in clinical trials—vorinostat, romidepsin, and belinostat (Table 3). Phase I/II trials have reported limited vorinostat activity in NSCLC but have shown promise when combined with other antineoplastic drugs, namely chemo- and immunotherapy [54–56]. Ongoing trials are exploring the efficacy of romidepsin and belinostat in lung cancer, with some early-phase studies that are also showing potential benefits in combination regimens. In a phase I trial, romidepsin showed activity in NSCLC when combined with erlotinib but several combination as well as single-agent studies are ongoing [57]. Early-phase studies are exploring belinostat in combination with other therapies, showing potential in enhancing anticancer effects [58].

**Table 3:** Recent clinical trials involving histone deacetylase inhibitors

NCT number and study title	Study status	Conditions	Interventions	Phases
**NCT01310244** MTD study PXD-101 in combination with paclitaxel + carboplatin in chemotherapy-naive patients with stage IV NSCLC	COMPLETED	NSCLC	Belinostat	PHASE I
			Carboplatin	PHASE II
			Paclitaxel	
**NCT02638090 Pembro and vorinostat for patients with stage IV Non-small Cell Lung Cancer (NSCLC)**	ACTIVE NOT RECRUITING	NSCCL	Vorinostat Pembrolizumab	PHASE I
				PHASE II
**NCT00128102 Suberoylanilide hydroxamic acid (Vorinostat, MK-0683) *Vs*. placebo in advanced malignant pleural mesothelioma (MK-0683-014)**	COMPLETED	Mesothelioma	Vorinostat	PHASE III
**NCT01059552 Treatment of locally advanced non-small cell lung cancer**	COMPLETED	Locally Advanced NSCLC	Vorinostat	PHASE I
**NCT00702962 Carboplatin and etoposide in combination with vorinostat for patients with extensive stage small cell lung cancer**	TERMINATED	SCLC	Vorinostat, Carboplatin, Etoposide	PHASE I
				PHASE II
**NCT00798720 Vorinostat and bortezomib as third-line treatment in advanced non-small cell lung cancer**	COMPLETED	NSCLC	Vorinostat	PHASE II
			Bortezomib	
**NCT00821951 Vorinostat in combination with palliative radiotherapy for patients with non-small cell lung cancer**	COMPLETED	NSCLC	Vorinostat Radiotherapy	PHASE I
**NCT04357873 Efficacy of immunotherapy plus a drug in patients with progressive advanced mucosal cancer of different locations**	ACTIVE NOT RECRUITING	Squamous Cell Cancer including Lung Cancer	Pembrolizumab; Vorinostat	PHASE II
**NCT00138203 Suberoylanilide hydroxamic acid in treating patients with stage iiib, stage IV, or recurrent non-small cell lung cancer**	COMPLETED	Recurrent and stage IV NSCLC	Vorinostat	PHASE II
**NCT01638533 Romidepsin in treating patients with lymphoma, chronic lymphocytic leukemia, or solid tumors with liver dysfunction**	ACTIVE NOT RECRUITING	Haematologic malignancies and solid cancers including recurrent Lung Carcinoma	Romidepsin	PHASE I
**NCT01302808 Romidepsin and erlotinib hydrochloride in treating patients with stage iii or stage iv non-small cell lung cancer**	COMPLETED	NSCLC	Erlotinib plus Romidepsin	PHASE I
**NCT00020202 FR901228 in treating patients with refractory or progressive small cell lung cancer or non-small cell lung cancer**	COMPLETED	Extensive stage SCLC	DRUG: FR901228	PHASE II
		Recurrent or stage IV NSCLC		
**NCT00094978 Depsipeptide/flavopiridol infusion for cancers of the lungs, esophagus, pleura, thymus or mediastinum**	TERMINATED	SCLC	Depsipeptide	PHASE I
		NCLC	Flavopiridol (alvocidib)	
**NCT00037817 Phase I study of gene induction mediated by sequential decitabine/depsipeptide infusion with or without concurrent celecoxib in subjects with pulmonary and pleural malignancies**	COMPLETED	SCLC	Decitabine	PHASE I
		NSCLC	Depsipeptide	
		Cancers of non-thoracic origin with metastases to the lungs or pleura	Celecoxib	

The next group of epigenetic drugs is bromodomain and extra-terminal domain inhibitors (BETis). BET inhibitors target bromodomains, which are involved in reading acetylated lysine residues on histones, leading to transcriptional repression of oncogenes like MYC [59]. Downregulation of MYC oncogene expression leads to growth inhibition in lung cancer cell lines. There are early-phase ongoing clinical trials, with some evidence of antitumor activity in solid tumor patients, including those with lung cancer—a phase I trial showed manageable toxicity and preliminary signs of antitumor activity, with further studies needed to confirm efficacy [60–62].

In lung cancer cell lines, BET inhibitors have been demonstrated to suppress cell proliferation and trigger apoptosis by downregulating MYC [63]. Animal studies have demonstrated that BETis can reduce tumor burden and enhance the effects of other treatments [64].

Finally, there are histone methyltransferase inhibitors (HMTis). Tazemetostat targets EZH2, a histone methyltransferase often overexpressed in cancer, including lung cancer [65,66]. The association between EZH2 overexpression and poor outcomes in lung cancer patients has been established [67]. Preclinical studies have shown that EZH2 inhibition can reduce tumor growth and metastasis by reversing EZH2-mediated gene silencing [68,69]. Clinical trials that are evaluating its efficacy in various cancers, including both SCLC and NSCLC, have yielded modest results. It remains to be evaluated whether a combination with checkpoint inhibitors could exceed them (Table 4).

**Table 4:** Recent clinical trials involving histone methyltransferase inhibitors

NCT number and study title	Study status	Conditions	Interventions	Phases
**NCT05467748** EZH2 Inhibitor, tazemetostat, and PD-1 blockade for treatment of advanced non-small cell lung cancer	NOT YET RECRUITING	NSCLC	Tazemetostat	PHASE I
			PD-1 inhibitor	PHASE II
**NCT05353439** Testing of tazemetostat in combination with topotecan and pembrolizumab in patients with recurrent small cell lung cancer	SUSPENDED	SCLC	Pembrolizumab Tazemetostat Topotecan	PHASE I
**NCT03874455** Tazemetostat expanded access program for adults with solid tumors	NO LONGER AVAILABLE	Solid Tumors including NSCLC	Tazemetostat	
**NCT03879798 DS-3201b and irinotecan for patients with recurrent small cell lung cancer**	TERMINATED	SCLC	DS-3201b	PHASE I
			Irinotecan	PHASE II

### Epigenetic therapy in combination treatments

The combination of DNA methylation inhibitors with other neoplastic agents, including chemotherapy, targeted therapy, as well as immunotherapy, has demonstrated encouraging outcomes. The rationale is that demethylating agents can sensitize cancer cells to these treatments by reactivating genes involved in apoptosis and immune response, potentially leading to improved outcomes [70,71].

Studies have indicated that combining DNMT inhibitors with chemotherapy drugs like cisplatin can enhance the cytotoxic effects on lung cancer cells [12,13]. This combination leads to increased DNA damage and apoptosis, providing a synergistic effect.

Interestingly, epigenetic therapy can also modulate the tumor microenvironment to enhance immune responses. DNMT inhibitors can increase the expression of immune-related genes and antigens, making tumor cells more recognizable to the immune system. This has prompted research into the combination of DNMT and immune checkpoint inhibitors in lung cancer [12,13].

The ability of epigenetic drugs to modulate the expression of genes and enhance the efficacy of other treatments provides a strong rationale for their continued development and integration into lung cancer treatment regimens.

### Targeting specific methylation changes

Identifying specific aberrantly methylated genes in lung cancer as well as developing targeted therapies against these changes is another promising approach. For example, restoring the function of hypermethylated tumor suppressor genes namely CDKN2A, MGMT, as well as RASSF1A could inhibit tumor growth and progression [12,13].

Re-expression of CDKN2A through demethylation can restore cell cycle control and inhibit proliferation. This has been demonstrated to improve the effectiveness of chemotherapy as well as targeted therapies in lung cancer models [12,13].

Demethylating MGMT can enhance the repair of the damage to the DNA caused by alkylating agents, improving response to these chemotherapeutic drugs in lung cancer treatment [12,13].

## Challenges and Future Directions

While the potential of methylation biomarkers for the detection of lung cancer in its early stages is promising, several challenges remain. There is a need for standardized protocols and assays to ensure consistent and reproducible results across different laboratories and clinical settings. The absence of standardized assay procedures can result in variability in results, complicating data comparison across trials and hindering the implementation of these biomarkers in routine clinical practice [3]. Extensive validation studies are required in order to confirm the clinical utility of these biomarkers across diverse populations and across different stages of lung cancer. Such studies would help establish the sensitivity, specificity, and overall diagnostic accuracy of methylation biomarkers, ensuring they can be reliably used for early detection and risk stratification in various demographic and clinical settings [41].

Furthermore, combining methylation markers with other biomarkers, such as genetic mutations and protein expression profiles, may enhance diagnostic accuracy and provide a more comprehensive understanding of the disease. This multi-modal approach can integrate different layers of biological information, potentially improving the sensitivity and specificity of lung cancer detection [12].

Costs attributed to the use of technologies for advanced methylation detection could be a barrier to widespread clinical adoption. Efforts should be made to develop cost-effective assays and improve accessibility, particularly in low-resource settings. Reducing costs and simplifying assay procedures would make these technologies more feasible for large-scale screening programs and routine clinical use [12].

Finally, therapeutic targeting of DNA methylation changes in lung cancer holds significant promise. By reversing aberrant methylation, combining epigenetic therapy with other treatments, and developing novel epigenetic drugs, it is possible to improve patient outcomes.

While DNA methylation patterns may have the potential to enhance our ability to diagnose lung cancer in its early stages, optimize its management, and deliver better patient outcomes, they are currently not a part of routine clinical practice. Only by overcoming these challenges, could we bring ourselves closer to harnessing the full potential of DNA methylation biomarkers in the detection of lung cancer and its optimal management. Continued research and clinical trials are essential in order to validate these approaches and integrate them into standard lung cancer treatment protocols [12,13].

## Conclusion

DNA methylation patterns offer a promising non-invasive method for the diagnosis of lung cancer in its early stages. Advances in methylation detection technologies and the identification of key biomarkers have the potential to revolutionize lung cancer screening, diagnosis, and management. Continued research and clinical validation are essential to fully realize the benefits of these epigenetic markers and integrate them into routine clinical practice, ultimately improving early detection rates, patient outcomes, and survival.

In summary, DNA methylation is a critical epigenetic modification linked to cancer development and progression. Both hypermethylation and hypomethylation contribute to the oncogenic process by altering genomic stability as well as the expression of genes. In lung cancer, these methylation changes offer valuable insights for diagnosis in the early stages of the disease, prognosis, and management as well as the creation of innovative therapeutic approaches. Understanding and targeting these epigenetic alterations hold promise for improving lung cancer outcomes.

## Data Availability

Data sharing is not applicable to this article as no datasets were generated or analyzed during the current study.
